# Modern Hybrid Rye, as an Alternative Energy Source for Broiler Chickens, Improves the Absorption Surface of the Small Intestine Depending on the Intestinal Part and Xylanase Supplementation

**DOI:** 10.3390/ani11051349

**Published:** 2021-05-10

**Authors:** Janine Donaldson, Sylwester Świątkiewicz, Anna Arczewka-Włosek, Siemowit Muszyński, Sylwia Szymańczyk, Marcin Bartłomiej Arciszewski, Anna Zacharko Siembida, Katarzyna Kras, Jose Luis Valverde Piedra, Tomasz Schwarz, Ewa Tomaszewska, Piotr Dobrowolski

**Affiliations:** 1 School of Physiology, Faculty of Health Sciences, University of the Witwatersrand, 7 York Road, Parktown, Johannesburg 2193, South Africa; 2 Department of Animal Nutrition and Feed Science, National Research Institute of Animal Production, 1 Krakowska St., 32-083 Balice, Poland; sylwester.swiatkiewicz@izoo.krakow.pl (S.Ś.); anna.arczewska@izoo.krakow.pl (A.A.-W.); 3 Department of Biophysics, Faculty of Environmental Biology, University of Life Sciences in Lublin, 13 Akademicka St., 20-950 Lublin, Poland; siemowit.muszynski@up.lublin.pl; 4 Department of Animal Physiology, Faculty of Veterinary Medicine, University of Life Sciences in Lublin, 12 Akademicka St., 20-950 Lublin, Poland; sylwia.szymanczyk@up.lublin.pl (S.S.); ewaRST@interia.pl (E.T.); 5 Department of Animal Anatomy and Histology, University of Life Sciences in Lublin, 20-950 Lublin, Poland; mb.arciszewski@wp.pl (M.B.A.); asiembida13@wp.pl (A.Z.S.); katarzyna.kras12@gmail.com (K.K.); 6 Department of Pharmacology, Toxicology and Environmental Protection, University of Life Sciences, Lublin, 13 Akademicka St., 20-950 Lublin, Poland; jose.valverde@up.lublin.pl; 7 Department of Animal Genetics, Breeding and Ethology, Faculty of Animal Sciences, University of Agriculture in Kraków, 24/28 Mickiewicza Ave., 30-059 Cracow, Poland; rzschwar@cyf-kr.edu.pl; 8 Department of Functional Anatomy and Cytobiology, Faculty of Biology and Biotechnology, Maria Curie-Sklodowska University, 19 Akademicka St., 20-033 Lublin, Poland

**Keywords:** broiler chickens, rye, xylanase, small intestine absorptive surface

## Abstract

**Simple Summary:**

The use of rye in poultry diets is associated with impairments in poultry performance due to the increased content of non-starch polysaccharides (NSPs). The performance of the body also depends on its health status, which can be reflected in structural changes of organs and systems. The gastrointestinal tract is the primary site of possible interaction between various nutrients, so changes in nutrition may be reflected in intestinal morphology. New hybrid rye varieties have been developed with a lower content of NSPs. Xylanase supplementation attenuates the negative effects of NSPs on poultry performance. The current study evaluated the inclusion of modern hybrid rye and xylanase supplementation on the absorptive surface of the small intestine of broilers. The results of the current study showed that the inclusion of modern hybrid rye to a corn–wheat-based diet improved the absorptive surface of the small intestine of broilers, regardless of xylanase supplementation.

**Abstract:**

The current study investigated the effects of the inclusion of modern hybrid rye (Brasetto variety) to a corn–wheat-based diet, with or without xylanase, on the absorptive surface of the small intestine of broilers. A total of 224 one-day-old male Ross 308 broiler chicks were randomly divided into four experimental groups with seven replicate cages of eight birds/replicate. A 2 × 2 factorial study design was used, with rye inclusion (0% or 20%) and xylanase supplementation (0 or 200 mg/kg of feed) as factors. Inclusion of rye increased duodenal and ileal crypt depth, villi height, the villus-to-crypt ratio and absorption surface area (*p* < 0.05), and ileal mucosa thickness and crypt width (*p* < 0.05). Xylanase supplementation attenuated the effects of rye in the duodenum and ileum and decreased the villi height and villus-to-crypt ratio in the jejunum (*p* < 0.05). Rye and xylanase had no effect on the spatial distribution of claudin 3 and ZO-1 protein, but xylanase supplementation reduced the amount of claudin 3 in the duodenum and jejunum (*p* < 0.05). The findings of this study indicate that 20% inclusion of modern hybrid rye to the diets of broilers improved the structure of the duodenum and ileum, but these effects were attenuated by xylanase supplementation.

## 1. Introduction

Corn is the most common energy source used in the diets of intensively reared poultry [1,2]. However, in an attempt to keep up with feed demands, alternative energy sources, including wheat, barley and rye have been investigated. Rye (*Secale cereale*) is resistant to fungal diseases, has a high tolerance to low temperatures, droughts and irregular soil pH and has a high production yield [3]. Nonetheless, the utilization of rye in poultry diets has been shown to impair poultry performance [4,5,6] due to its high content of non-starch polysaccharides (NSPs), mainly in the form of arabinoxylans [7]. The limited capacity of monogastric animals to digest these NSPs results in increased digesta viscosity, decreased nutrient digestibility, slowed passage of the digesta through the gastrointestinal tract and the increased incidence of wet and sticky droppings [8,9,10].

To attenuate the deleterious effects of NSPs, poultry feeds are routinely supplemented with exogenous xylanases, which hydrolyze the complex carbohydrates (NSPs) present in cereal cell walls and consequently decrease digesta viscosity and release nutrients which can then be utilized by the animal [2,11]. The inclusion of xylanase to poultry diets has been shown to improve overall performance indices [2,11,12]. Strategies to improve feed efficiency are particularly important for environmental and economic sustainability, and increasing investments and efforts are made to decrease antinutritional factors in cereal varieties. Recently, new hybrid varieties of rye have been developed, with a lower NSP content, specifically with regards to that of arabinoxylans [13,14,15]. Conflicting results with regards to the effect of xylanase supplementation on the intestinal histomorphometry of broiler chickens, specifically with regards to goblet cell number, villous height and crypt depth, have been observed [16,17]. Thus, it was hypothesized that more complex structural indices combined with epithelial integrity traits would indicate the true effect of modern hybrid rye on broiler small intestine. Therefore, the objective of the current study was to investigate the effects of the inclusion of hybrid rye (of the Brasetto variety) in corn–wheat-based diets, with or without xylanase supplementation, on the intestinal absorptive surface through the assessment of intestinal histomorphometry and the expression and quantification of tight junction proteins (zonula occludens and claudin-3) in broiler chickens.

## 2. Materials and Methods

### 2.1. Birds and Experimental Diets

This study is part of a larger project on modern cereals in poultry and livestock farming. It provides new and additional data and analysis from the perspective of structural changes in the broiler digestive system from previously reported studies [1,11]. A completely randomized study on 224 one-day-old male Ross 308 broiler chicks was performed at the Experimental Station of the National Research Institute of Animal Production in Balice, Poland. Chicks, with an average weight of 41 g, were randomly divided into four experimental treatments, with seven replicate cages of eight birds/replicate. A 2 × 2 factorial study design was used, with rye inclusion (0% or 20%) and xylanase supplementation (0 or 200 mg/kg of feed) as factors. The birds were kept in a wire-floored battery cage with 7800 cm^2^ total floor space in the cage (length × width × height = 120 × 65 × 50 cm), in an environmentally controlled room in the poultry house. During the study, the temperature in the experimental facility was maintained from 32 °C at 1 d of age to 21 °C at 21 d of age and later, relative humidity cycled from 50 to 60%, air exchange was 1 m^3^/1 kg of body weight gain (BWG)/1 h and concentrations of CO_2_ and NH_3_ were maintained below 2000 and 20 ppm, respectively. Each cage was equipped with four nipple-cup drinkers and a trough feeder along the front of the cage. Water and feed were supplied ad libitum. Feed was supplemented ad libitum as follows: starter (1 to 21 D), and grower-finisher (22 to 42 D) feeding phases. Table 1 presents the composition and calculated nutrient contents of the experimental diets.

Nutrient requirements of broilers were met or exceeded, according to poultry feeding standards, by formulated isonitrogenous and isoenergetic experimental diets [19]. The content of non-starch polysaccharides divided into soluble and insoluble fractions along with the content of arabinoxylans in rye grain and experimental diets was analyzed by gas chromatography as previously described [20] (Table 2).

The hybrid winter rye cv. Brasetto was used as a partial replacement of corn in the diet. In order to improve the high NSP levels within the rye, xylanase Ronozyme WX (DSM Nutritional Products Sp. z o.o., Mszczonów, Poland) with a minimum xylanase activity of 1000 FXU/g was used as a supplement to the diet. As previously stated, the chemical composition of the rye grain included 9.23% of crude protein, 0.81% of crude fat, 1.46% of crude fiber and 1.51% of crude ash [11].

### 2.2. Tissue Collection and Histomorphometry Analysis

At the end of the experiment, birds were weighed, and seven representative birds (one per replicate cage with body weight close to the average) from each group were slaughtered by decapitation after electrical stunning, following a 12-h fast. During the necropsy, two 20-mm-long samples of the duodenum (2 cm after stomach), jejunum (from 50% of intestine length) and ileum (2 cm before ceca), were collected and processed for standard histology, as previously described [21,22]. The segments were gently cut open longitudinally along the mesentery line and placed flat (not stretched) into a standard histological cassette. Tissues were fixed in 4% buffered formaldehyde (pH 7.0) for 24 h, washed in tap water and dehydrated in graded ethanol solutions. Samples were further trimmed to achieve four transverse macro sections (4 × 8 mm in dimensions, transverse section on the longer side) from each individual and were processed in Ottix Plus and Ottix Shaper (DiaPath, Martinengo, Italy) with the use of a tissue processor (STP 120, Thermo Scientific, Waltham, MA, USA) to saturate the samples in paraffin. Paraffin blocks were then made using an embedding station (MYR EC-350, Casa Álvarez Material Científico S.A. Madrid, Spain). Two paraffin blocks were made for each chicken for each intestinal segment, containing two 4 × 8 mm tissue samples. The tissue samples were placed to represent sections at successive depths of the intestine 4 mm apart. Twenty cross sections (with 20 µm intervals between each five-slice section), 5 µm thick, were then cut with a microtome (Microm HM 360, Microm, Walldorf, Germany) from each small intestine paraffin block (80 cross sections/bird/intestine segment in total). The samples were stained with Goldner’s trichrome, and bright field microscopic (two-dimensional) images (magnification ×100, ×200) were taken with a confocal microscope (AXIOVERT 200 M, Carl Zeiss, Jena, Germany) and a color digital camera (AxioCam HRc, Carl Zeiss, Jena, Germany) for histology and histomorphometry analysis of intestine structures [21,22,23]. For each bird and intestinal segment, 20 cross-sections were selected that showed the course of the sampled intestinal segment and did not duplicate the assessed structures with respect to villi and crypts. Twelve measurements of each parameter evaluated were taken at each of the selected cross sections. The multiple measurements were averaged. The following parameters were analyzed: the thickness of the serous membrane (magnification ×200), the outer and inner muscle layer (magnification ×100), the submucosa, the lamina muscularis mucosa (magnification ×200) and the mucosa; crypt width (measured in the middle of the crypt depth) and depth (defined as the depth of the invagination between adjacent villi, from the bottom of the crypt to the base of the villus), as well as villi height (from the tip of the villus to the villus–crypt junction) and width (measured in the middle of the villus height) (magnification 100); (measurements were taken on villi and crypt assemblies) absorption surface area and villus length-to-crypt depth ratio, as previously described [22,24]. Histomorphometry measurements were made with the use of graphic analysis software (ImageJ 1.53, National Institutes of Health, Bethesda, MD, USA; available at: http://rsb.info.nih.gov/ij/index.html, accessed on 20 November 2020).

### 2.3. Immunohistochemistry

Immunohistochemical staining was performed on the tissue slices according to a previously described protocol [25]. Heat-induced epitope retrieval was performed using a pressure cooker Rapid Cook (Morphy Richards, Swinton, UK) in sodium citrate buffer (10 mM sodium citrate, 0.05% Tween 20, pH 6.0). Endogenous peroxidase activity was blocked subsequently with a 3% solution of hydrogen peroxide in deionized water for 5 min. After blocking for 30 min in normal serum, sections were incubated with the first antibodies over-night at 4 °C. Two types of antibodies were used to mark tight junctions: rabbit polyclonal anti-ZO-1 (zonula occludens -1) antibody (orb11587, Biorbyt, St. Louis, MO, USA, dilution 1:500) and rabbit polyclonal anti-claudin 3 antibody (AB15102; Abcam, Cambridge, UK, dilution 1:100). The sections were then incubated for 30 min with the second antibody (peroxidase conjugated goat anti-rabbit, #611-1302, Rockland Immunochemicals, Inc. Limerick, PA, USA, dilution 1:500). Negative control sections for each antibody were obtained by identical immunohistochemical staining, excluding the primary antibody. The sections were then developed in 3,3′-diaminobenzidine tetrahydrochloride (DAB D5905; Sigma–Aldrich, St. Louis, MO, USA) used as chromogens, for 15 min at room temperature. Counterstaining was performed with Mayer’s hematoxylin (MHS32-1L; Sigma–Aldrich, St. Louis, MO, USA).

### 2.4. Western-Blot Analysis

Tissue samples obtained from relevant segments, adjacent to the above-mentioned histology samples of the small intestine, were collected from every sacrificed chicken and were used for Western-Blot analysis, as previously described previously [26]. Briefly, the samples were homogenized in lysis buffer (125 mM TRIS-HCl pH 6.8; 4% SDS; 10% glycerol; 100 mM DTT), boiled in a water bath for 10 min and centrifuged at 10,000× *g* for 10 min. The supernatant was then collected. The protein content was determined using the Bradford method [27]. Samples containing 80 µg of protein were separated by 10% SDS-PAGE and then electroblotted onto an Immobilon P membrane (Sigma–Aldrich, St. Louis, MO, USA). The membranes were blocked with 3% low fat milk in PBS for 1 h, after the transfer, and incubated overnight with polyclonal rabbit anti-ZO-1 (orb11587, Biorbyt, St. Louis, MO, USA, dilution 1:1000) or rabbit polyclonal anti-claudin 3 (AB15102; Abcam, Cambridge, UK, dilution 1:1000) antibodies. The membranes were washed three times for 10 min with PBS containing 0.05% TRITON X-100 (Sigma–Aldrich, St. Louis, MO, USA) and incubated for 2 h in the presence of the second antibody (peroxidase conjugated goat anti-rabbit, #611-1302, Rockland Immunochemicals, Inc. Limerick, Pennsylvania, USA, dilution 1:10,000). The membranes were visualized with 3,3′-diaminobenzidine tetrahydrochloride (DAB D5905; Sigma–Aldrich, St. Louis, MO, USA). An anti-β-actin antibody (1:2000, Sigma–Aldrich, St. Louis, MO, USA) was used as the loading control. Densitometry analysis of the obtained blots was performed. A gel analysis function was used to determine the rows of blots and to determine the optimal level of background correction. A background subtraction function was then applied to compensate for background irregularities using the rolling ball algorithm built into ImageJ [28] to determine appropriate thresholds for blot marking. The blots were densitometrically quantified and normalized to their corresponding β-actin bands. Western-Blot analysis was performed in triplicate, and an average for each bird was used for statistical analysis.

### 2.5. Statistical Analysis

Data were analyzed by 2-way ANOVA using the GLM procedure of Statistica 13 (TIBCO Software Inc., Palo Alto, CA, USA). The model included diet and xylanase and their interactions as fixed effects. The replicate cage served as the experimental unit. Tukey’s test was used to compare differences between means when the model was declared significant (*p* < 0.05). The sample size was calculated for a two-sided test with an α of 0.05 and power at 0.8, with an effect size of 1.63 [29].

## 3. Results

### 3.1. Changes in Intestinal Histomorphometry

Results from the histomorphometry measurements are presented in Table 3, Table 4 and Table 5. The morphology of the duodenum, jejunum and ileum was affected by both the rye inclusion and xylanase supplementation. Significant main effects of both additives, as well as their interactions, were noted.

Duodenal histomorphometry parameters are presented in Table 3. The crypt depth (17, 11 and 9%, all *p* < 0.001) and villi height (25, 20 and 12%, all *p* < 0.001) were increased in the duodenum of broilers receiving rye or xylanase, or both rye and xylanase in the feed compared to broilers not receiving rye and xylanase in the feed. In both cases, the effect of rye was attenuated by xylanase (*p* < 0.001). The absorption surface area was increased in broilers receiving rye in the feed (29%, *p* < 0.001), and the villus-to-crypt ratio was increased in broilers receiving rye (7%, *p* = 0.025) or xylanase (8%, *p* = 0.005) compared to birds without rye and xylanase in the feed. In addition, in both these cases, the effect of rye was attenuated by xylanase, especially with respect to the villi-to-crypt ratio (*p* < 0.001). There was no response to feed with rye or xylanase or both in other parameters such as serous membrane thickness, outer and inner muscle layer, submucosa and lamina muscularis mucosa (*p* > 0.05). It was shown that the main effect of the addition of xylanase was a reduction in the thickness of the inner muscle layer by 16%. Although there were no differences between groups for serosa and mucosa thickness, the interaction of rye and xylanase still existed, and xylanase attenuated the response of these individual duodenal elements to rye in the feed (Table 3).

Jejunal histomorphometry parameters are presented in Table 4. Feed containing rye and xylanase caused 6% (*p* < 0.001) increase of jejunal crypts depth compared to groups with xylanase supplementation and without rye or xylanase, whereas 9% (*p* < 0.001) increase compared to broilers receiving 20% rye in the feed. Unlike in the duodenum, xylanase stimulated deepening of crypts when the feed contained rye. Villi height and villus-to-crypt ratio were reduced by 10% (*p* = 0.002) and 15% (*p* < 0.001), respectively, in broilers receiving rye and xylanase in the feed compared to broilers without rye or xylanase in the feed. The observed effect could be strengthened by additive impact of rye, since these two parameters were lowered by 4 and 5% respectively by the main effect of rye content (Table 4). The addition of xylanase caused reduction of villi height by 7% (*p* = 0.026), villi width by 20% (*p* = 0.019) and villus-to-crypt ratio by 7% (*p* = 0.009), compared to broilers without xylanase in the feed. The thickness of the serosa, outer and inner muscle layer, submucosa, lamina muscularis mucosa, mucosa, crypt width and absorption surface area were not affected by the rye inclusion or xylanase supplementation (*p* > 0.05). Although rye, xylanase, or both had no effect on mucosal thickness compared with broilers not receiving rye or xylanase in the feed, xylanase exacerbated the lowering effect of rye.

Ileal histomorphometry parameters are presented in Table 5. The feed with 20% rye inclusion increased the thickness of the mucosa by 10% (*p* = 0.004), crypt width by 10% (*p* < 0.001) and depth by 19% (*p* < 0.001), villi height by 13% (*p* < 0.001), absorption surface area by 23% (*p* < 0.001) and villous to crypt ratio by 12% (*p* = 0.020) compared to feed without this cereal and xylanase. Xylanase addition to the broiler feed increased the crypt depth by 19% (*p* < 0.001), villi height by 13% (*p* = 0.006) and villi width by 15% (*p* = 0.009) compared to the broilers receiving feed without rye and xylanase. The effect of rye inclusion depended on the addition of xylanase in all the parameters mentioned above. Xylanase attenuated the response of the intestinal mucosa, crypts and intestinal villi to rye. The absorption surface area and the ratio of villous to crypt, increased by rye inclusion, were also decreased by xylanase. Only crypt depth was stimulated by both rye inclusion and xylanase addition but without additive effect in broilers receiving rye and xylanase in the feed (Table 5). No changes were noted in the thickness of the serous membrane, outer and inner muscle layers, submucosa and the lamina muscularis mucosa.

### 3.2. Epithelial Barrier Characteristics (Expression and Quantification of Tight Junction Proteins)

**Figure 1:**
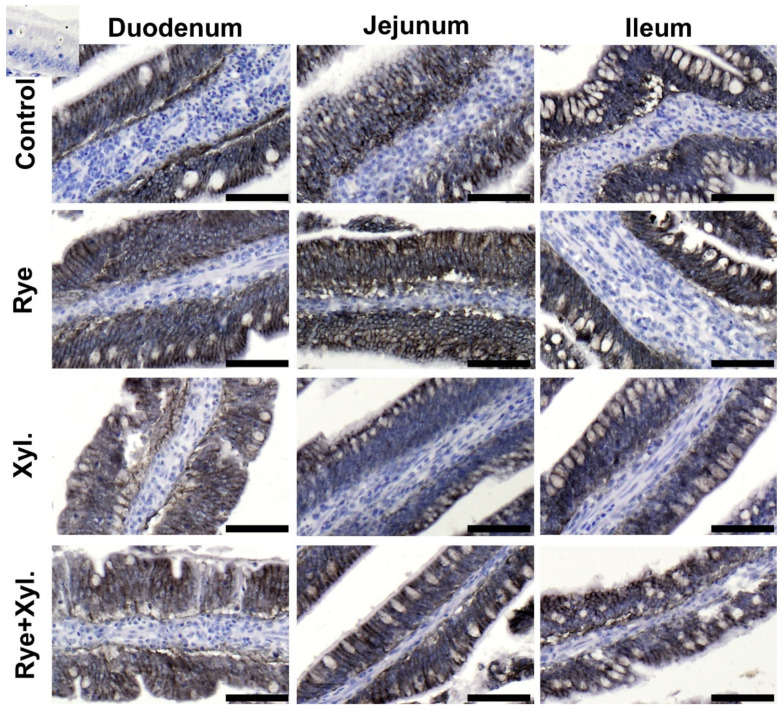
Epithelial expression and spatial distribution of Claudin 3 in the villi epithelium of the assessed intestine part. Control (0% rye inclusion, 0 mg/kg of feed xylanase supplementation), Rye (20% rye inclusion, 0 mg/kg of feed xylanase supplementation), Xyl. (0% rye inclusion, 200 mg/kg of feed xylanase supplementation), Rye + Xyl. (20% rye inclusion,200 mg/kg of feed xylanase supplementation). Black staining localizes the examined protein. Upper left corner insert—antibody control. Scale bars represent 100 µm.

The immunolocalization of claudin 3 and ZO-1 (zonula occludens tight junction protein-1) proteins was performed through microscopic observation of the small intestine epithelium of the duodenum, jejunum and ileum. The immune reactions with antibodies in the examined parts of the digestive tract were similar in all treatment groups. The reactions were continuous, and claudin 3 and ZO-1 were observed on the pericellular borders of the epithelium cells. The spatial distribution of the proteins assessed was normal, without any signs of epithelial gaps. There were no visible differences in the intensity of protein expression between treatment groups (Figure 1).

**Figure 2:**
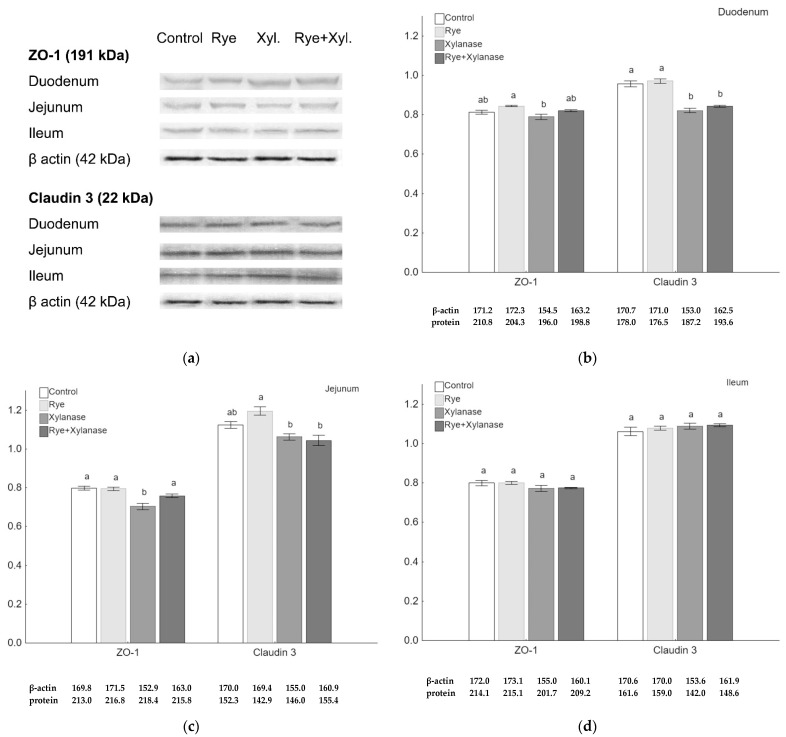
Protein expression in the epithelium of selected sections of the small intestine of broiler chickens. (**a**) Representative Western-Blots of examined proteins in the intestine epithelium obtained from broilers receiving no rye or xylanase in the feed (0% rye, 0 mg/kg xylanase), rye group (20% rye inclusion), xylanase-supplemented group (200 mg/kg of feed) and rye + xylanase (20% rye inclusion and xylanase 200 mg/kg of feed); (**b**–**d**) The relative abundance of ZO-1 and claudin 3, expressed as the mean ratio relative to β-actin of the respective sections of the small intestine; whiskers represent the SEM. Different letters (**a**–**c**) above the bars show differences at *p* < 0.05.

Quantitative analysis of claudin 3 and ZO-1 proteins was performed using the Western-Blot method. Although there were no visible differences in the immunohistochemistry microscopic assessment, the quantitative analysis of the ZO-1 amount showed a decrease in the jejunum of broilers receiving xylanase (0.70) compared to broilers receiving feed with rye and xylanase (0.76) (*p* = 0.037), feed with rye (0.79) (*p* = 0.002) and feed without rye and xylanase (0.80) (*p* = 0.002) (Figure 2c). Rye normalized ZO-1 protein levels when broilers were fed with xylanase (*p* = 0.036). Lower amounts of the ZO-1 protein were also detected in the duodenal epithelium of broilers receiving xylanase in the feed (0.78) compared to broilers receiving feed with 20% rye inclusion (0.84) (*p* = 0.028) (Figure 2b). The amount of claudin 3 was significantly decreased in the duodenum of broilers receiving xylanase (0.82) and rye with xylanase (0.84) compared to the broilers receiving no rye and xylanase in the feed (0.96) and rye inclusion (0.97) (*p* < 0.001 for both comparisons) (Figure 2b). The amount of claudin 3 was higher in the jejunum of broilers receiving rye (1.19) in the feed compared to those receiving xylanase (1.06) (*p* = 0.010) and broilers receiving rye and xylanase (1.04) (*p* = 0.004) (Figure 2c). There were no differences between groups in the amounts of ZO-1 and claudin 3 in the ileum (Figure 2d).

## 4. Discussion

The current study investigated the effects of the inclusion of modern hybrid rye (Brasetto variety) to a corn–wheat-based diet, with or without xylanase supplementation, on the absorptive surface of the small intestine of broilers. Previous studies have shown that the use of rye, as an alternative energy source in poultry diets, has been associated with increased viscosity of the digesta and thus poor digestibility and absorption of ingested nutrients [8]. Rye inclusion, as well as xylanase supplementation, has yielded conflicting results with regards to their effects on the absorptive surface of the small intestine.

The small intestine is the primary site for the digestion and absorption of ingested nutrients [30,31]. Some of the digestive processes and all of the absorptive processes take place in or around the intestinal villi and crypts [32]. In fact, villus height and crypt depth, along with villus height-to-crypt depth ratio and villi absorptive surface area, are key parameters which define the functional capacity of the small intestine [33,34,35]. Various dietary nutrients or additives have been shown to affect the morphology of the intestinal mucosa, and in doing so affect overall nutrient metabolism [33,36,37].

In the current study, both the rye inclusion and the xylanase supplementation affected the morphology of the small intestine, with the duodenum and ileum being the most affected, followed by the jejunum. The inclusion of rye in the broiler’s diet had a more pronounced effect on the small intestine histomorphometry compared to the xylanase supplementation. The enzyme-induced changes in villus height, crypt depth and villus height-to-crypt depth ratio in the small intestine of broilers in the current study contrast with previous findings. Wu et al. (2004) observed no significant effects of xylanase supplementation on the villus height, crypt depth and villus height-to-crypt depth ratio in the duodenum, jejunum and ileum of broilers fed a wheat-based diet supplemented with xylanase (1000 XU/kg diet), for a period of 21 days [17]. Similarly, Yan et al. (2017) also observed no significant changes in mid-duodenal and mid-gut villus height, crypt depth and villus height-to-crypt depth ratio following 21 days of carbohydrase supplementation (at 0.05%, providing 1000 units of 1,4-β-xylanase per kg diet) to broilers fed a basal diet containing rye, soft wheat, soybean meal and feather meal [4]. Pekel, Horn and Adeola (2017) also observed no effects of xylanase supplementation (800 xylanase units/kg of diet) on the jejunum villus height or crypt depth of broilers fed corn–soybean meal-based diets for a period of 14 days [38]. Souza et al. (2014) observed a decrease in the crypt depth and an increase in the villus height-to-crypt depth ratio in the ileum of laying hens fed diets based on corn and soybean meal, following xylanase supplementation (100 g/tonne of feed, equivalent to an enzyme activity of 16,000 BXU/kg) for a period of four weeks [39]. Since villus morphology is dependent on the enteral absorption of nutrients, the inconsistencies among previous studies involving exogenous enzyme supplementation may be due to the type of cereal used in the diets or the overall diet composition [38,40].

Dietary inclusion of the new hybrid Brasetto rye significantly increased the duodenal crypt depth and villus-to-crypt ratio, as well as the ileal villus height of the broilers in the current study. Since the duodenum is the primary site for nutrient absorption [30], it would be expected that this section of the small intestine of broilers in the current study was the most affected in terms of the diet-induced changes in intestinal morphology. An increase in villus height or in villus height-to-crypt depth ratio has been associated with improved nutrient digestion and absorption [41,42]. The increased villus-to-crypt ratio and increased villus height observed in the duodenum and ileum of broilers in the current study, following dietary rye inclusion, could possibly suggest an improvement in the overall capacity of the small intestine for the digestion and absorption of ingested nutrients, as confirmed by the significant increase in the duodenal and illeal absorption surface area. As the base of the crypts is constantly dividing to maintain the structure of the villi, an increase in crypt depth would produce more developed villi [43]. The increase in the duodenal and illeal crypt depth of broilers in the current study could be indicative of increased proliferative activity within the intestinal mucosa in the region of the villi, allowing for renewal of the villi if necessary and ensuring improved digestion and absorption of the nutrients consumed [44,45]. Previous studies have yielded varying results with regards to the effects of dietary rye inclusion on intestinal morphometry, which were mostly dependent on the section of small intestine examined and the composition of the basal diet to which the rye was added. El-Wahab et al. (2020) observed no significant changes in ileal morphological parameters of broilers following the addition of increasing amounts of rye (5%, 10%, 20%, 30%) to a wheat and soybean meal-based control diet [46]. Van Krimpen, Torki and Schokker, (2017) observed an increase in villus height and crypt depth in the jejunum of broilers following the inclusion of 5% or 10% rye to a maize-based diet from d 14 to 28 [5].

In addition to its digestive and absorptive functions, the intestine also serves as an important barrier against the external environment, preventing the entry of toxins, food antigens and harmful microorganisms [47,48,49]. Intestinal barrier dysfunction is associated with altered tight junction protein expression [50]. Tight junctions are the connections between enterocytes which are responsible for regulating the paracellular diffusion of solutes and ions across the intestinal epithelium [51,52]. To assess the integrity of the intestinal barrier of broilers in the current study, the expression of tight junction proteins, claudin-3 and zonula occludens-1, was assessed. The immune reactions with antibodies against the tight junction proteins in the examined parts of the tract were not affected by rye inclusion or xylanase supplementation, with the spatial distribution of the proteins being evenly distributed thorough epithelium and no signs of any gaps within the epithelium barrier. There were also no visible differences in the intensity of protein expression between treatment groups. With regards to the quantitative analysis of claudin-3 and zonula occludens-1, neither the rye inclusion nor the xylanase supplementation had a significant effect on the amount of claudin-3 and zonula occludens-1 in the ileum of broilers in the current study. However, xylanase supplementation decreased the amount of zonula occludens-1 protein in both the duodenum and jejunum and the amount of claudin-3 protein in the duodenum. These findings are not in agreement with previous studies involving the use of xylanase in pigs, which, like birds, are monogastric animals. He et al. (2020) observed increased gene expression of zonula occludens-1 in the intestine of piglets supplemented with 30 or 60 mg/kg (24,000, 48,000 BXU/kg, respectively) of xylanase for 28 days [53]. Tiwari et al. (2018) also observed increased concentrations of claudin, occludin and zonula occludens-1 in the jejunum of piglets fed a corn–soybean meal-based diet supplemented with xylanase (1500 endo-pentosanase unit of xylanase/kg of the diet) [54]. Thus, the effects of xylanase supplementation on tight junction protein expression and in turn the intestinal barrier function need to be further explored.

The present study has its limitation since physiological approach and methods would provide greater insight into the effects of rye and xylanase on the nutrient absorption rate and would allow the establishment of intestinal barrier permeability and integrity in the context of rye and xylanase interaction. It would also be an interesting approach to study different doses of xylanase considering the present findings on the effects of the rye and xylanase interaction. The present study, however, indicates that modern hybrid rye varieties can be used as a safe energy source for broiler chickens from the standpoint of structural integrity and small intestinal function.

## 5. Conclusions

In conclusion, the current study showed that modern hybrid rye of the Brasetto variety can be added to corn–wheat-based diets for broiler chickens, as an alternative energy source, without adversely affecting small intestine morphology. In fact, dietary rye inclusion has the potential to improve the structure of the small intestine and to increase the absorptive surface, but xylanase supplementation attenuates these effects.

## Figures and Tables

**Table 1:** Composition (g/kg) and nutritive value of the experimental diets *.

Item	Starter ^1^ (Day 1 to 21)		Grower-Finisher ^2^ (Day 22 to 42)	
	Corn–Wheat	Corn–Wheat–Rye	Corn–Wheat	Corn–Wheat–Rye
Rye	0	200	0	200
Corn	457.1	302.1	404.4	238.4
Wheat	100	40	200	150
Soybean meal	370	370	304	307
Rapeseed oil	33	48	52	67
Monocalcium phosphate	15	15	13	13
Limestone	13.5	13.5	14	14
Sodium chloride	3	3	3	3
Premix vit./min.	5 ^1^	5 ^1^	3 ^2^	3 ^2^
DL-methionine, 99%	2.6	2.6	2.3	2.3
L-lysine HCl, 99%	0.8	0.8	1.7	1.7
L-threonine, 98.5%	-	-	0.6	0.6
Thr (%)	0.85	0.85	0.81	0.81
Val (%)	1.04	1.04	0.95	0.95
Calculated composition s ^3^				
Metabolizable energy (MJ/kg)	12.6	12.6	13.1	13.1
Crude protein (%)	22.2	22.2	20.5	20.5
Lysine (%)	1.23	1.23	1.15	1.15
Methionine (%)	0.58	0.58	0.56	0.56
Total calcium (%)	0.97	0.97	0.93	0.93
Total phosphorus (%)	0.71	0.71	0.66	0.66
Available phosphorus (%)	0.45	0.45	0.41	0.41
Total calcium/available phosphorus	2.16	2.16	2.27	2.27

^1^ The premix provided per 1 kg of starter diet: Mn, 100 mg; Zn, 100 mg; Fe, 50 mg; Cu, 20 mg; I, 1 mg; Se, 0.35 mg; vitamin A, 12,500 IU (retinol); vitamin D3, 1.25 mg (cholecalciferol), vitamin E, 137 IU (dl-alphatocopherol); vitamin K3, 3 mg (menadione); vitamin B1, 3 mg (thiamine); vitamin B2, 8 mg(riboflavin); vitamin B6, 4 mg (pyridoxine); vitamin B12, 0.02 mg (cyanocobalamin); biotin, 0.2 mg; folic acid, 2 mg; niacin, 50 mg; Ca-pantothenate, 16.3 mg; choline chloride, 348 mg; coccidiostat:100 ppm of total narasin/nicarbazin activity. ^2^ The premix provided per 1 kg of grower-finisher diet: Mn, 80 mg; Zn, 90 mg; Fe, 40 mg; Cu, 20 mg; I, 0.5 mg; Se, 0.2 mg; vitamin A, 10,000 IU (retinol); vitamin D3, 0.75 mg (cholecalciferol); vitamin E, 33 IU (dl-alphatocopherol); vitamin K3, 2.5 mg (menadione); vitamin B1, 2.5 mg (thiamine); vitamin B2, 5 mg (riboflavin); vitamin B6, 3.51 mg (pyridoxine); vitamin B12, 0.021 mg (cyanocobalamin); biotin, 0.201 mg; folic acid, 1.0 mg; niacin, 35 mg; Ca-pantothenate, 13 mg; choline chloride, 300 mg; coccidiostat: narasin 70 ppm. ^3^ Calcuated according to the European Table [18] as a sum of the ME content of components. * Adapted from Muszyński et al. and Arczewska-Włosek et al. [1,11]. “-“ not applicable.

**Table 2:** Non-starch polysaccharide (NSP) content of rye grain and experimental diets (g/kg DM) ^1^.

Item	Rye Grain	Starter		Grower-Finisher	
		Corn–Wheat	Corn–Wheat–Rye	Corn–Wheat	Corn–Wheat–Rye
Total NSP	155.0	109.5	99.1	105.4	108.6
Soluble NSP	55.0	16.9	19.7	12.8	16.8
Arabinoxylans	85.5	37.5	36.5	41.1	42.9

^1^ Adapted from Arczewska-Wlosek et al. [11].

**Table 3:** Histomorphometric properties of the duodenum of male, Ross 308 broiler chicks following the 42-day experimental period.

Treatment and Effects	Serous Membrane Thickness, µm	Thickness of the Outer Muscle Layer, µm	Thickness of the Inner Muscle Layer, µm	Thickness of the Submucosa, µm	Thickness of the Lamina Muscularis Mucosa, µm	Thickness of the Mucosa, µm	Crypts Width, µm	Crypts Depth, µm	Villi Height, µm	Villi Width, µm	Absorption Surface µm^2^	Villus/ Crypt Ratio
Main effect rye content												
0%	16.4	26.3	86.3	3.7	15.7	1225.2	35.2	101.9	966.1	127.1	18.9	9.5
20%	17.2	24.8	78.8	3.7	15.7	1225.7	34.3	106.3	1043.1	117.8	21.5	9.5
Main effect xylanase addition												
0 mg/kg	17.0	25.7	89.9	3.8	16.9	1232.5	34.9	104.9	988.8	120.7	19.8	9.4
200 mg/kg	16.6	25.4	75.2	3.6	14.5	1218.4	34.6	106.3	1020.4	124.1	20.5	9.6
Treatment effect												
0% + 0 mg/kg	15.0	27.0	95.6	3.8	17.0	1199.2	35.0 ^a^	96.6 ^c^	879.9 ^c^	124.9	17.3 ^b^	9.1 ^b^
0% + 200 mg/kg	17.8	25.5	77.0	3.6	14.4	1251.3	35.4 ^a^	107.2 ^b^	1052.3 ^a^	129.3	20.5 ^ab^	9.8 ^a^
20% + 0 mg/kg	19.0	24.4	84.3	3.8	16.8	1265.8	34.8 ^ab^	113.3 ^a^	1097.7 ^a^	116.6	22.4 ^a^	9.7 ^a^
20% + 200 mg/kg	15.4	25.3	73.4	3.7	14.5	1185.5	33.8 ^b^	105.4 ^b^	988.5 ^b^	119.0	20.6 ^ab^	9.4 ^ab^
Pooled SEM	1.48	1.02	5.86	0.17	1.28	29.95	0.28	0.80	13.7	5.47	0.58	0.14
Main effects and interaction												
rye content	0.591	0.176	0.213	0.811	0.987	0.989	0.002	<0.001	<0.001	0.099	<0.001	0.581
xylanase addition	0.809	0.775	0.018	0.339	0.064	0.642	0.271	0.095	0.028	0.567	0.229	0.146
(rc) × (xa)	0.038	0.267	0.515	0.881	0.917	0.035	0.017	<0.001	<0.001	0.859	<0.001	<0.001

Data presented are the means (*n* = 7 for treatment effect); a, b, c—mean values in columns with different letters differ significantly at *p* < 0.05; SEM—standard error of the means. (rc) × (xa)—interaction between the rye content (rc) and xylanase addition (xa).

**Table 4:** Histomorphometric properties of the jejunum of male, Ross 308 broiler chicks following the 42-day experimental period.

Treatment and Effects	Serous Membrane Thickness, µm	Thickness of the Outer Muscle Layer, µm	Thickness of the Inner Muscle Layer, µm	Thickness of the Submucosa, µm	Thickness of the Lamina Muscularis Mucosa, µm	Thickness of the Mucosa, µm	Crypts Width, µm	Crypts Depth, µm	Villi Height, µm	Villi Width, µm	Absorption Surface, µm^2^	Villus/ Crypt Ratio
Main effect rye content												
0%	8.5	22.5	65.7	4.7	14.8	1102.7	28.2	85.5	782.7	67.7	23.6	9.2
20%	8.8	22.8	64.0	4.4	14.2	1010.1	28.1	86.7	751.5	75.1	22.4	8.7
Main effect xylanase addition												
0 mg/kg	8.5	22.6	65.5	4.7	14.4	1048.3	27.9	84.1	790.5	76.1	22.7	9.4
200 mg/kg	8.8	22.7	64.2	4.4	14.7	1064.5	27.8	88.1	743.6	66.8	22.7	8.4
Treatment effect												
0% + 0 mg/kg	8.2	22.4	66.8	4.8	15.0	1060.9 ^ab^	28.1	85.1 ^bc^	812.3 ^a^	75.1 ^a^	23.4	9.5 ^a^
0% + 200 mg/kg	8.7	22.6	64.6	4.5	14.7	1144.4 ^a^	28.3	85.9 ^b^	753.0 ^b^	60.3 ^b^	23.7	8.8 ^b^
20% + 0 mg/kg	8.7	22.7	64.3	4.5	13.7	1035.7 ^b^	27.8	83.1 ^c^	768.7 ^ab^	77.0 ^a^	22.0	9.3 ^ab^
20% + 200 mg/kg	9.0	22.8	63.7	4.4	14.7	984.6 ^b^	28.4	90.3 ^a^	734.3 ^b^	73.3 ^a^	22.8	8.1 ^c^
Pooled SEM	0.36	1.41	3.46	0.34	0.69	27.82	0.16	0.65	13.85	3.32	0.68	0.16
Main effects and interaction												
rye content	0.334	0.882	0.623	0.065	0.338	0.002	0.781	0.077	0.032	0.033	0.707	0.007
xylanase addition	0.331	0.922	0.696	0.062	0.669	0.564	0.800	<0.001	0.002	0.009	0.969	<0.001
(rc) × (xa)	0.717	0.962	0.815	0.356	0.350	0.022	0.568	<0.001	0.377	0.109	0.661	0.293

Date presented are the means (*n* = 7 for treatment effect); a, b, c—mean values in columns with different letters differ significantly at *p* < 0.05; SEM—standard error of the means. (rc) × (xa)—interaction between the rye content (rc) and xylanase addition (xa).

**Table 5:** Histomorphometric properties of the ileum of male, Ross 308 broiler chicks following the 42-day experimental period.

Treatment and Effects	Serous Membrane Thickness, µm	Thickness of the Outer Muscle Layer, µm	Thickness of the Inner Muscle Layer, µm	Thickness of the Submucosa, µm	Thickness of the Lamina Muscularis Mucosa, µm	Thickness of the Mucosa, µm	Crypts Width, µm	Crypts Depth, µm	Villi Height, µm	Villi Width, µm	Absorption Surface, µm^2^	Villus/ Crypt Ratio
Main effect rye content												
0%	5.7	41.9	135.9	4.7	22.7	810.2	27.0	85.8	608.5	77.1	17.8	7.1
20%	6.0	40.9	125.4	4.7	21.2	832.4	29.2	93.2	683.8	70.7	19.8	7.4
Main effect xylanase addition												
0 mg/kg	6.0	40.9	127.9	4.7	22.0	823.0	28.5	85.6	664.6	73.0	19.3	7.3
200 mg/kg	5.7	41.9	133.4	4.8	21.9	819.5	27.8	93.5	627.8	74.8	18.3	6.7
Treatment effect												
0% + 0 mg/kg	5.7	40.5	135.9	4.6	23.2	784.1 ^b^	27.1 ^c^	78.3 ^b^	571.9 ^c^	71.8 ^b^	17.3 ^b^	7.3 ^b^
0% + 200 mg/kg	5.6	43.3	135.8	4.9	22.3	836.2 ^ab^	26.9 ^c^	93.4 ^a^	645.2 ^b^	82.5 ^a^	18.3 ^b^	6.9 ^bc^
20% + 0 mg/kg	6.2	41.4	119.8	4.7	20.8	862.0 ^a^	29.8 ^a^	92.9 ^a^	757.3 ^a^	74.3 ^ab^	21.3 ^a^	8.2 ^a^
20% + 200 mg/kg	5.7	40.5	131.0	4.7	21.6	802.8 ^b^	28.6 ^b^	93.6 ^a^	610.4 ^bc^	67.2 ^b^	18.4 ^b^	6.5 ^c^
Pooled SEM	0.19	1.87	5.42	0.19	1.60	14.33	0.21	0.78	14.36	2.18	0.47	0.19
Main effects and interaction												
rye content	0.091	0.623	0.063	0.786	0.341	0.132	<0.001	<0.001	<0.001	0.006	<0.001	0.260
xylanase addition	0.095	0.625	0.317	0.564	0.964	0.806	0.002	<0.001	0.016	0.423	0.047	<0.001
(rc) × (xa)	0.908	0.324	0.307	0.582	0.581	0.001	0.043	<0.001	<0.001	<0.001	<0.001	0.003

Date presented are the means (*n* = 7 for treatment effect); a, b, c—mean values in columns with different letters differ significantly at *p* < 0.05; SEM—standard error of the means. (rc) × (xa)—interaction between the rye content (rc) and xylanase addition (xa).
